# Identification of novel citramalate biosynthesis pathways in *Aspergillus niger*

**DOI:** 10.1186/s40694-019-0084-7

**Published:** 2019-11-19

**Authors:** Abeer H. Hossain, Aiko Hendrikx, Peter J. Punt

**Affiliations:** 1 Dutch DNA Biotech B.V., Padualaan 8, 3584 CH Utrecht, The Netherlands; 2 Molecular Biology and Microbial Food Safety, Swammerdam Institute for Life Sciences, University of Amsterdam, Science Park 904, 1098 XH Amsterdam, The Netherlands

**Keywords:** Itaconic acid biodegradation, *Aspergillus niger*, Transcriptome analysis, Metabolic engineering, Citramalate synthase, Citramalate, Organic acid transport

## Abstract

**Background:**

The filamentous fungus *Aspergillus niger* is frequently used for industrial production of fermentative products such as enzymes, proteins and biochemicals. Notable examples of industrially produced *A. niger* fermentation products are glucoamylase and citric acid. Most notably, the industrial production of citric acid achieves high titers, yield and productivities, a feat that has prompted researchers to propose *A. niger* to serve as heterologous production host for the industrial production of itaconic acid (IA), a promising sustainable chemical building-block for the fabrication of various synthetic resins, coatings, and polymers. Heterologous production of IA in *A. niger* has resulted in unexpected levels of metabolic rewiring that has led us to the identification of IA biodegradation pathway in *A. niger*. In this study we have attempted to identify the final product of the IA biodegradation pathway and analyzed the effect of metabolic rewiring on the bioproduction of 9 industrially relevant organic acids.

**Results:**

IA biodegradation manifests in diminishing titers of IA and the occurrence of an unidentified compound in the HPLC profile. Based on published results on the IA biodegradation pathway, we hypothesized that the final product of IA biodegradation in *A. niger* may be citramalic acid (CM). Based on detailed HPLC analysis, we concluded that the unidentified compound is indeed CM. Furthermore, by transcriptome analysis we explored the effect of metabolic rewiring on the production of 9 industrially relevant organic acids by transcriptome analysis of IA producing and WT *A. niger* strains. Interestingly, this analysis led to the identification of a previously unknown biosynthetic cluster that is proposed to be involved in the biosynthesis of CM. Upon overexpression of the putative citramalate synthase and a genomically clustered organic acid transporter, we have observed CM bioproduction by *A. niger*.

**Conclusion:**

In this study, we have shown that the end product of IA biodegradation pathway in *A. niger* is CM. Knock-out of the IA biodegradation pathway results in the cessation of CM production. Furthermore, in this study we have identified a citramalate biosynthesis pathway, which upon overexpression drives citramalate bioproduction in *A. niger*.

## Background

The filamentous fungus *Aspergillus niger* is widely known for its secretion capacity of metabolites, proteins and enzymes. Due to the species robust nature, and range of interesting compounds with generally regarded as safe (GRAS) status that it can produce, *A. niger* is a frequently used work-horse in industrial biotechnology [1]. Notable examples of industrial compounds produced by *A. niger* are citric acid, oxalic acid, gluconic acid, amylase and glucoamylase [2–5]. *A. niger* has also been proposed for the production of heterologous products, such as cyclodepsipeptides, a class of secondary metabolites that exhibit a variety of pharmaceutically relevant bioactivities and itaconic acid (IA), a promising sustainable chemical building-block for the fabrication of various synthetic resins, coatings, and polymers [6, 7].

The production of IA with *A. niger* reaches industrially relevant titers due to an rewired pathway involving the endogenous cytosolic citrate synthase CitB and ATP-citrate lyase [8, 9]. Together with an improved fermentation protocol this resulted in the highest IA titer reported for *A. niger* (56.5 g/l) [9]. However, this metabolic rewiring towards IA proved more intricate as we have also observed induction of genes that are responsible for IA bioconversion and degradation in high IA producing *A. niger* strains [10]. The gene products itaconyl-CoA transferase (IctA) and itaconyl-CoA hydratase (IchA) together constitute a pathway that bears much similarity with IA degrading pathways reported in *A. terreus* and the pathogenic bacteria *Pseudomonas aeruginosa* and *Yersinia pestis* [11, 12]. However, whereas the IA degrading pathways in aforementioned species convert IA into the cellular building-blocks pyruvate and acetyl-CoA, the end product of the pathway in *A. niger* is unknown, as the gene encoding the enzyme that facilitates the final step in the conversion of citramalyl-CoA into pyruvate and acetyl-CoA, *cclA*, although present, is not induced in *A. niger* under IA degrading conditions. We have previously reported that an unknown peak was detected during HPLC analysis in samples where extracellular IA titers were diminishing [10] Deletion of the pathway specific genes *ictA* and *ichA* results in cessation of IA bioconversion and concomitantly the unknown peak is also no longer detected [10]. In this study, we have focused on identifying the gene pathways related to this unknown compound, identified as citramalate (CM), and its link with IA production in *A. niger*. To further explore the unexpected level of metabolic rewiring in IA producing *A. niger* strains, we have analyzed the transcriptome of high and low IA producing strains for genes that are related to biosynthesis and transport of the industrially relevant metabolites citric acid, succinic acid, fumaric acid, malic acid, lactic acid, gluconic acid, oxalic acid, itaconic acid and citraconic acid to see the effects on these genes, which interestingly have led to the identification of another completely unknown CM biosynthesis route [13].

## Materials and methods

### Strains and culture conditions

All *A. niger* strains used in this study are listed in Table 1. All strains are maintained on minimal medium (MM) plates (10 g/l glucose, 16 g/l agar, 6 g/l NaNO_3_, 0.52 g/l KCl, 1.52 g/l KH_2_PO_4_, 0.0022 g/l ZnSO_4_ × 7H_2_O, 0.0011 g/l H_3_BO_3_, 0.0005 g/l MnCl_2_ × 4H_2_O, 0.0005 g/l FeSO_4_ × 7H_2_O, 0.00017 g/l CoCl_2_ × 6H_2_O, 0.00016 g/l CuSO_4_ × 5H_2_O, 0.00015 g/l NaMoO_4_ × 2H_2_O, 0.005 g/l Na_2_EDTA and 0.5 g/l Mg_2_SO_4_), or liquid complete medium (LCM) (MM + 2.5 g/l yeast extract). The medium was supplemented with 2.44 g/l uridine and 1.12 g/l uracil (UU) when required. Typically, plates were incubated at 35 °C, MTPs were incubated at 33 °C, and shake flasks were incubated at 35 °C. Spore suspensions were prepared by harvesting spores from MM plates after 3–5 days incubation at 35 °C using physiological salt solution (0.9% NaCl) and subsequent filtering of the solution through Miracloth (EMD Millipore). For long term storage strains were stored in 20% glycerol at − 80 °C.

**Table 1:** Strains used in this study

Strain	Abbreviation	Description
AB1.13	AB1.13 WT	Uridine auxotroph [14]
AB1.13 *pyrG*+	AB1.13	Uridine prototroph of AB1.13 [15]
AB1.13 CAD 4.1	AB1.13 CAD	Selected pyrG+ transformant of *cadA* expressing transformant (CAD10.1) of AB1.13 [16]
AB1.13 CAD + MFS + MTT #49B;	AB1.13 #49B	Selected *mttA* expressing transformants of AB1.13 CAD + MFS 3.9 [8]
AB1.13 CAD + MFS + MTT + CitB #99	CitB#99	Selected *citB* overexpressing strain of AB1.13 CAD + MFS + MTT #49B [8]
AB1.13 cimA A10 AB1.13 cimA B3 AB1.13 cimA D11	CimA A10 CimA B3 CimA D11	Selected *cimA* overexpressing strain of AB1.13 (this study)
AB1.13 cimA B3 pyrE−	CimA B3 pyrE−	*pyrE* mutant strain of CimA #B3 (this study)
AB1.13 cimA B3 + mfsB 27	CimA + MfsB #27	Selected *mfsB* overexpressing strain of AB1.13 cimA B3 pyrE− (this study)
AB1.13 cimA B3 + mfsB 28	CimA + MfsB #28	

### Auxotrophic mutant (*pyrE*) selection

CimA #B3 was cultivated on MM agar plates in the presence of 5-Fluoroorotic acid (5-FOA) to generate *pyrE* mutant strains, resulting in uridine auxotrophy. Spores of colonies were transferred to MM + 5-FOA agar supplemented with uridine and uracil in 48 well plates, using sterilized toothpicks, for an additional selection round. Growing strains of the second selection round were transferred to MM without uridine and uracil to check if the 5-FOA resistant mutants were indeed uridine auxotroph. DNA was isolated from uridine auxotrophic transformants, as described in “Vector construction and transformation”, and PCR with primers 98 + 99 (Additional file 1: Table S1) was performed to confirm *pyrE* mutant strains.

### Vector construction and transformation

Restriction digestion, ligation and other standard molecular biological techniques were performed using common procedures [17]. All primers were obtained from Eurogentec and are listed in Additional file 1: Table S1. PCR reactions were performed with the Alpha Cycler 4 (PCRmax). All enzymes were purchased from ThermoFisher and used following the manufacturer’s protocols. Fungal DNA isolations for colony PCR were performed on mycelia grown in 1 ml LCM in a 2 ml round well 96-well microtiter plate (MTP) (Axygen) sealed with semi permeable film at 33 °C, 850 rpm, overnight in a rotary shaker. DNA was isolated from the mycelia using the DNA isolation from Plant kit and protocol (Nexttec GmbH). This included homogenization with 300 μl acid washed 0.1 mm Zirconium beads (Biospec Products) and 2 × 1 min bead-beating with cooling on ice in between (Mini-Beadbeater-96). The supernatant was directly used as template for PCR.

To create overexpression construct of *cimA* the An09g00170 gene was in vitro synthesized at GeneArt (Waltham, MA) and subsequently digested with HindIII. The digested *cimA* fragment was ligated in HindIII digested pAB*gpdI* vector containing the *A. niger gpdA* expression signals, thereby establishing the pABgpdI-cimA expression vector.

For the construction of an *mfsB* (An09g00190) expression vector, *mfsB* was PCR amplified from AB1.13 genomic DNA with Phusion HF Master Mix and primer pair 432 + 433 (Additional file 1: Table S1) following standard protocols. The resulting fragment was purified, using the QIAquick PCR purification kit and protocol (Qiagen). 2.5 μg of the purified fragment was digested with NcoI/BpiI in one reaction, and BpiI/BglII in a second reaction. The 1123 bp generated fragment from the first reaction and 623 bp generated fragment from the second reaction were excised from gel and purified using the QIAquick gel extraction kit and protocol (Qiagen). These two fragments were inserted into an NcoI/BglII opened pAB-gpdI backbone carrying the *gpdA* expression signals, originating from pABgpdI-citC [10], establishing the *mfsB* expression vector pABgpdI-mfsB. This was done in a ligation reaction consisting of T4 DNA ligase and buffer, and a total of 140 μg DNA with a vector:insert ratio of 1:3. The mixture was incubated at room temperature for 30 min. 4 μl of the ligation mixture was transformed into *Escherichia coli* JM109 (Promega) according to the manufacturer’s standard heat shock protocol. Presence of pABgpdI-mfsB in colonies was checked with colony PCR, using DreamTaq Green PCR Master Mix. Several positive transformants were miniprepped according to the GeneJET Plasmid Miniprep kit and protocol (ThermoFisher). Restriction analysis with BpiI was performed to validate the plasmids identity, followed by maxiprep of designated transformants using the Plasmid Plus Maxi kit and protocol (Qiagen). The identity of the purified plasmid was verified by Sanger sequencing using primers 143, 329, 430 and 431 (Additional file 1: Table S1) (Baseclear).

For transformation of *A. niger*, linear DNA fragments were used. The linear DNA fragment PgpdA-cimA-TgpdA and PgpdA-mfsB-TgpdA was obtained through PCR amplification with Phusion HF Master Mix, and primers 80 + 81 (Additional file 1: Table S1). These PCR fragments were co-transformed with linear fragments of PpyrE-pyrE-TpyrE (2.7 kb) (for *mfsB* overexpression) and with pAB4-1, that harbours the *A. niger pyrG* gene for (*cimA* overexpression) [18], in an ratio of 1:10 (0.5 µg marker:5 µg construct).

Transformants were plated on MM + 1.2 M sorbitol as osmotic agent and selected based on the reestablishment of uracil prototrophy due to integration of the functional *pyr* expression cassette. Individual colonies were transferred to 48-well plates containing MM agar. These were used to inoculate MTPs, and DNA was isolated as described earlier. Successful integration of the *mfsB* and *cimA* expression cassettes was determined by colony PCR, using primers 143 + 433 for PgpdA-mfsB-TgpdA and 143 + 331 for PgpdA-cimA-TgpdA. Positive transformants were streaked on MM plates to obtain pure colonies. DNA isolation and colony PCR was repeated as described above, and positive transformants were used to prepare spore suspensions as described in “Strains and culture conditions”.

### Shake flask cultivations

Shake flask production tests were performed in 300 ml non-baffled shake flasks containing 60 ml or 500 ml non-baffled shake flasks containing 100 ml M12++ medium (1.43 g/l NH_4_NO_3_, 0.11 g/l KH_2_PO_4_, 0.5 g/l MgSO_4_ × 7H_2_O, 0.005 g/l CuSO_4_ × 5H_2_O, 0.0006 g/l FeCl_3_ × 6H_2_O, 0.0006 g/l ZnSO_4_ × 7H_2_O, 0.074 g/l NaCl, 0.13 g/l CaCl_2_ × 2H_2_O and 100 g/l glucose, adapted from Li et al. [16]). Shake flasks were inoculated with 1 × 10^6^ spores/ml medium and incubated at 35 °C, 250 rpm for up to 2 weeks. 350 μl samples were taken daily, filtered and used to determine extracellular metabolite concentrations by HPLC as described in section “Metabolite analysis”. Flasks were weighed before sampling to correct metabolite concentrations for medium evaporation.

### Metabolite analysis

Extracellular metabolite concentrations were determined by high-performance liquid chromatography (HPLC). A WATERS e2695 separations module equipped with an Aminex HPX-87H column (Bio-Rad) was used in combination with 5 mM H_2_SO_4_ as eluent, coupled to a refractive index detector (WATERS 2414) and a dual-wavelength detector (WATERS UV/Vis 2489) for peak detection. For identification of various organic acids as described in fungal biosynthetic pathways, reference compounds were analyzed for retention time and UV210_nm_/RI area ratios. Empower PDA software was used for data processing.

### RNA isolation, transcriptome sequencing and analysis

Biomass samples for RNA isolation were taken at several timepoints during controlled-batch cultivation and washed with distilled water and frozen in liquid N_2_. The controlled batch cultivations were performed using 5 l scale benchtop New Brunswick Scientific fermenters (BioFlo 3000) at 33 °C. Starting pH was 3.5 after inoculation and M12 medium [16] was allowed to naturally acidify till pH 2.3 and then kept at pH 2.3 by addition of 4 M KOH. Dissolved oxygen (DO) tension was 25% at the moment of inoculation and when DO dropped till 20% it was kept at 20%. The system was calibrated with 100% sterile air as 100% DO and 100% N_2_ as 0% DO. The fermenter was inoculated by 72 h old 100 ml non-baffled shake flask cultures containing 1.0*10^8^ spores. mRNA isolation procedures for transcriptome sequencing and analysis are published in Hossain et al. [10].

## Results

### IA bioconversion in *A. niger* revisited

**Fig. 1:**
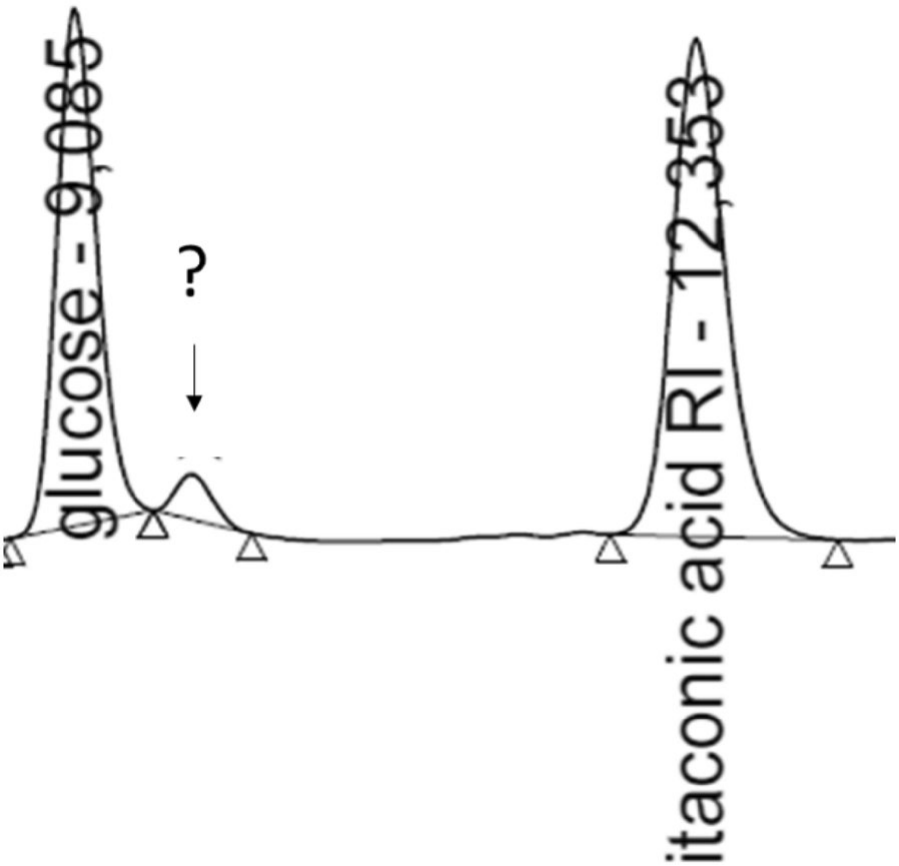
Unidentified peak next to the glucose peak on RI detector in sample after 14 days cultivation of CitB#99 performed in 500 ml non-baffled flasks [10]

In our previous communication, we have reported the role of *ictA* and *ichA* during IA bioconversion in *A. niger* [10]. We have observed that the expression of *ictA* and *ichA* is induced under IA producing conditions in high IA producing strain CitB#99 and knock-out of these genes resulted in abolishment of IA bioconversion [10]. Surprisingly, during IA bioconversion, we have also observed the occurrence of an previously unidentified compound in HPLC analysis (Fig. 1). Upon further analysis we hypothesized that this unidentified peak could be citramalic acid (CM), as based on the identified IA bioconversion pathway, citramalyl-CoA is an intermediate that is formed during IA bioconversion, which could be converted to CM by action of IctA [10, 11]. To confirm this, detailed HPLC analysis was carried out. Based on this analysis, the unidentified compound shared a very similar retention time and UV210_nm_/RI area ratio, as the CM standard (Additional file 1: Tables S2 and S3). Based on this observation we concluded that the unidentified peak is CM.

**Fig. 2:**
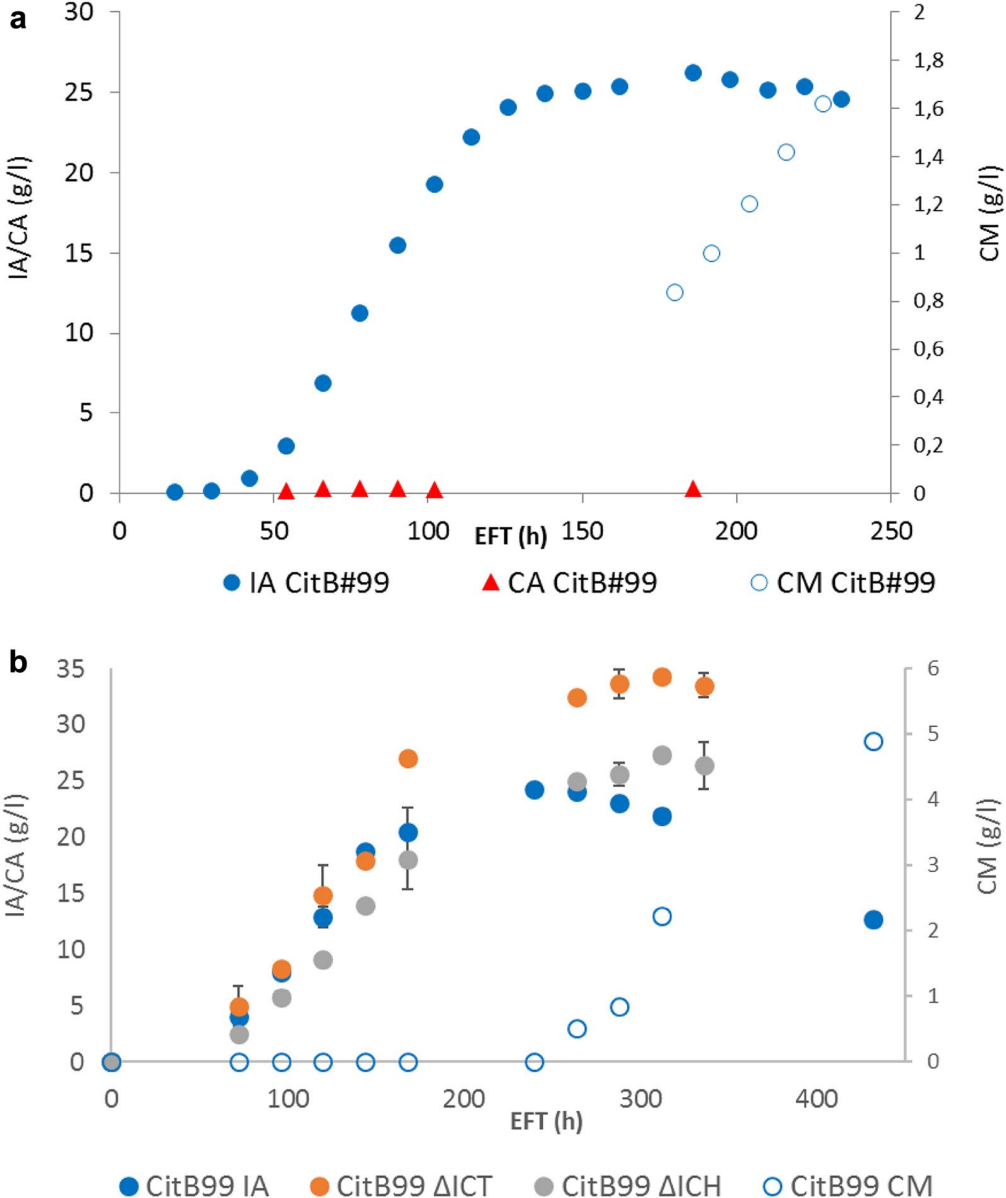
Previous fermentation data revisited. **a** Controlled batch-cultivation of CitB#99 in 5 l bioreactors [8]. CM can be detected after 180 h EFT when IA titers start to diminish. **b** Non-shaking 500 ml flask cultivation [10]. CM detected in cultivations with CitB#99 after 264 h EFT when IA titer starts to drop. No CM detected in cultivations with CitB#99 ΔICT and CitB#99 ΔICH

Upon this observation, we have revisited our previous fermentation data as presented in Hossain et al. [8, 10] to identify the presence of CM during conditions of IA bioconversion (Fig. 2). Interestingly, in both cases we have been able to identify the presence of CM in the extracellular medium upon decreasing levels of IA in the medium. The occurrence of CM in both cases coincides with reducing titers of IA, further strengthening the hypothesis that IA is converted into CM in *A. niger* (Fig. 2).

### Alternative organic acid production in *A. niger*

It is well established in literature that *A. niger* is a prolific producer of organic acids, more particular gluconic, citric and oxalic acid [19]. The unexpected finding of CM biosynthesis and secretion during IA bioconversion prompted us to search for additional and previously unidentified organic acid biosynthesis pathways in this organism. For this purpose we have explored the genome mining efforts presented by Li et al. [13] in *A. niger* to identify genes potentially related to the biosynthesis of nine industrially relevant organic acids: citric acid, succinic acid, fumaric acid, malic acid, lactic acid, gluconic acid, oxalic acid, itaconic acid and citraconic acid. In Table 2 we have summarized transcriptome data of these genes. Furthermore, transcriptome data of the putative orthologs and paralogs in *A. niger* of the well described transporters for malic acid (MaeA) [20], itaconic acid (Itp1 and MfsA) [8, 21, 22] and hydroxyparaconic acid (Itp1) [22] are presented together with the citrate exporter CexA [23] (Table 3). From this latter comparison it is interesting to note that the two functionally characterized IA transporters have different putative orthologs in *A. niger* (Table 3), which by itself is a IA non-producing strain.

**Table 2:** Transcriptome data of genes involved in the biosynthesis of industrially relevant organic acids

	New locus tag	Old locus tag	Gene product	Gene name	Localization	RPKM values			
						AB1.13	AB1.13 CAD	AB1.13 #49B	CitB#99
1	ANI_1_1206064	An07g09530	Pyruvate dehydrogenase E1 component subunit alpha		Mito	319.96	296.42	259.17	203.01
1	ANI_1_622094	An11g04550	Pyruvate dehydrogenase E1 component subunit alpha		Mito	18.21	16.89	23.09	27.18
1	ANI_1_12014	An01g00100	Pyruvate dehydrogenase E1 component subunit beta		Mito	204.86	158.00	136.09	125.15
2	ANI_1_274064	An07g02180	Pyruvate dehydrogenase E2 component		Mito	232.08	210.10	209.07	225.04
3	ANI_1_440184	An04g02090	Pyruvate carboxylase	*pycA*	Cyto	306.10	262.83	309.14	395.41
4	ANI_1_876084	An09g06680	Citrate synthase	*citA*	Mito	284.82	269.02	255.71	238.09
4	ANI_1_1474074	An08g10920	Citrate synthase	*citB*	Cyto	3.05	3.10	2.87	10838.65
4	ANI_1_2950014	An01g09940	Citrate synthase	*citC*	Cyto	463.10	370.88	438.08	96.44
5	ANI_1_470084	An09g03870	Aconitate hydratase		Mito	56.65	37.15	45.12	50.78
5	ANI_1_3018024	An02g11040	Aconitate hydratase		Cyto	0.04	0.04	0.00	0.00
5	ANI_1_1410074	An08g10530	Aconitate hydratase	*acoA*	Mito	326.50	284.14	426.11	486.32
5	ANI_1_1808144	An16g05760	Aconitate hydratase		Cyto	1.30	1.13	1.03	2.02
5	ANI_1_578044	An05g02230	Aconitate hydratase		Cyto	2.91	7.34	6.20	11.04
5	ANI_1_1802134	An15g07730	Aconitate hydratase		Cyto	28.75	43.79	46.11	27.81
6	ANI_1_906164	An18g06760	Isocitrate dehydrogenase (NAD+) subunit 1		Mito	189.71	209.47	167.93	190.84
6	ANI_1_798074	An08g05580	Isocitrate dehydrogenase [NAD] subunit 2		Mito	165.29	175.80	165.81	151.75
6	ANI_1_3136024	An02g12430	Isocitrate dehydrogenase [NADP]		Per	53.93	50.46	43.22	64.47
7	ANI_1_826184	An04g04750	2-Oxoglutarate dehydrogenase		Mito	115.53	103.38	107.27	88.87
8	ANI_1_1482094	An11g11280	Dihydrolipoyllysine-residue succinyltransferase		Mito	168.01	172.17	158.22	198.85
9	ANI_1_230154	An17g01670	Succinyl-CoA ligase [GDP-forming] subunit alpha		Mito	264.96	249.57	263.12	194.37
9	ANI_1_58124	An14g00310	Succinyl-CoA ligase [GDP-forming] subunit beta		Mito	277.86	256.78	249.99	212.81
10	ANI_1_1750024	An02g12770	Succinate dehydrogenase [ubiquinone] flavoprotein subunit		Mito	245.43	193.93	185.80	107.81
10	ANI_1_2706024	An02g07600	Succinate dehydrogenase [ubiquinone] flavoprotein subunit		Mito	0.47	0.35	0.79	0.95
11	ANI_1_952104	An12g07850	Fumarate hydratase		Mito	121.25	107.39	124.39	128.67
12	ANI_1_12134	An15g00070	Malate dehydrogenase	*mdhA*	Cyto	402.08	426.49	462.06	636.37
12	ANI_1_268064	An07g02160	Malate dehydrogenase		Mito	500.20	510.92	572.33	565.47
12	ANI_1_2230094	An11g07190	Malate dehydrogenase		Cyto	0.14	0.15	0.00	0.15
13	ANI_1_1256014	An01g09270	Isocitrate lyase	*acuD*	Per	86.38	61.77	65.20	42.26
13	ANI_1_1336134	An15g02980	Isocitrate lyase/malate synthase		Per	33.76	34.75	25.03	22.66
13	ANI_1_1826104	An12g05180	Isocitrate lyase/malate synthase		Cyto	0.00	0.09	0.00	0.48
14	ANI_1_320134	An15g01860	Malate synthase		Per	112.41	98.34	72.33	34.38
15	ANI_1_2114184	An04g08220	l-Lactate dehydrogenase		Mito/Cyto	0.09	0.58	0.51	0.63
16	ANI_1_1536084	An09g06220	PrpD-like protein		Cyto	0.30	0.23	0.36	0.43
16	ANI_1_2952014	An01g09950	PrpD-like protein		Cyto	552.06	456.98	383.21	165.41
16	ANI_1_2948014	An01g09930	PrpD-like protein		Mito	308.68	261.08	288.92	145.66
16	ANI_1_3352024	An02g14730	PrpD-like protein		Cyto	5.97	7.29	5.81	10.08
17	ANI_1_1432064	An07g00760	Itaconyl-CoA transferase	*ictA*	Mito	13.07	108.35	170.34	375.89
18	ANI_1_2118064	An07g09220	Itaconyl-CoA hydratase	*ichA*	Mito	7.32	60.28	146.61	232.65
19	ANI_1_1156014	An01g08610	Citramalate-CoA lyase	*cclA*	Mito	10.17	12.59	21.43	19.54
20	ANI_1_92174	An10g00820	Oxaloacetate acetylhydrolase	*oahA*	Cyto	1803.48	1122.69	323.44	9.81
20	ANI_1_2054064	An07g08390	Oxaloacetate acetylhydrolase		Mito	10.41	7.31	7.55	11.70
21	ANI_1_1678104	An12g03440	Glucose oxidase		Secreted	0.66	0.84	0.21	0.23
21	ANI_1_748094	An11g05580	Glucose oxidase		Secreted	1.01	1.80	1.15	0.19
21	ANI_1_1398064	An07g00450	Glucose oxidase		Secreted	1.47	0.67	0.38	1.45
21	ANI_1_1992014	An01g14740	Glucose oxidase	*goxC*	Secreted	1.25	0.70	0.56	0.20
22	ANI_1_106174	An10g00900	Gluconolactonase		Cyto	1.37	2.05	1.64	3.30
22	ANI_1_254044	An05g02030	Gluconolactonase		Cyto/Nucleus	1.75	1.79	1.91	2.98
22	ANI_1_1902144	An16g06620	Gluconolactonase		Secreted	0.00	0.00	0.00	0.00
23	ANI_1_928084	An09g00170	2-Isopropylmalate synthase	*cimA*	Cyto	362.80	298.38	108.92	23.52
24	ANI_1_440024	An02g03250	Isopropylmalate isomerase		Cyto	135.20	57.70	45.96	96.62

RPKM values, taken from Hossain et al. [10] were calculated according to the method presented by Mortazavi et al. [28] in order to normalize data for gene length. Protein localization was predicted using the WoLF PSORT algorithm (https://wolfpsort.hgc.jp/). For those cases where the gene listed has been studied in more detail the corresponding gene name was indicated

**Table 3:** Transcriptome data of putative orthologs and paralogs in *A. niger* to functionally characterized organic acid transporters in filamentous fungi

New locus tag	Old locus tag	Transporter	Protein sequence coverage (%)	Protein sequence similarity (%)	RPKM values			
					AB1.13	AB1.13 CAD	AB1.13 #49B	CitB#99
		Citrate transporter CexA *A. niger* (Steiger et al. [23])						
ANI_1_478154	An17g01710	Citrate transport protein (*cexA*)	100	100	57.83	44.83	189.54	58.61
		**Citramalate transporter MfsB** ***A. niger*** **(This study)**						
ANI_1_930084	An09g00190	MFS multidrug transporter (*mfsB*)	100	100	197.34	140.59	62.55	10.52
ANI_1_1618104	An12g03020	MFS multidrug transporter	96	62	0.99	1.05	2.50	8.32
		**Itaconate transporter MfsA** ***A. terreus*** **(Li et al. Hossain et al. [**8, 16**])**						
ANI_1_2702024	An02g07580	MFS transporter	84	36	2.81	2.52	2.07	2.42
		**Hydroxyparaconate transporter Itp1** ***Ustilago maydis*** **(Geiser et al. Hosseinpour et al. [**21, 22**])**						
ANI_1_478154	An17g01710	Citrate transport protein (*cexA*)	93	37	57.83	44.83	189.54	58.61
		**Malate transporter MaeA** ***A. oryzae*** **(Knuf et al. [**20**])**						
ANI_1_2040144	An16g08330	C4-dicarboxylate transporter/malic acid transport protein	97	71	1.32	1.95	1.75	1.54

RPKM values, taken from Hossain et al. [10] were calculated according to the method presented by Mortazavi et al. [28] in order to normalize data for gene length. Protein similarity and coverage scores were obtained using the BLAST algorithm (https://blast.ncbi.nlm.nih.gov/Blast.cgi?PROGRAM=blastp&PAGE_TYPE=BlastSearch&LINK_LOC=blasthome)

Interestingly, the expression of the canonical glucose oxidase *goxC*, that is responsible for gluconic acid formation, is practically absent in the analyzed strains under the cultivation conditions that we applied. Furthermore it is interesting to observe the significant downregulation of *oahA*, that encodes oxaloacetate hydrolase and is responsible for oxalate production, in high IA producing strain CitB#99 compared with AB1.13. Both results correspond with the consequent absence of gluconic acid and oxalic acid in HPLC analyses of cultivations with IA producing strains [8, 10].

Glyoxylate shunt specific genes An01g09270 and An15g01860 that code for isocitrate lyase (*acuD*) and malate synthase respectively are downregulated in CitB#99. It is well established in literature that itaconate can inhibit the glyoxylate shunt in pathogenic bacteria, however it was not known if this is also the case in fungi [12, 24, 25]. Our results suggest a relation of IA bioproduction and glyoxylate shunt downregulation.

Another interesting observation is that *citB* overexpression downregulated expression of another putative cytosolic citrate synthase *citC*, similar as *citB,* being part of a secondary metabolite pathway of which all genes are downregulated, including two cadA like genes An0g09950 and An01g09930 [10].

Furthermore, we have observed that the expression of a gene encoding a 2-isopropylmalate synthase (IPMS) like protein (An09g00170), with significant similarity to a bacterial citramalate synthase (*cimA*), is strongly reduced in CitB#99 [26]. However, the expression of An01g13160, that codes for the canonical IPMS, is not affected. This uncharacterized gene encoding the IPMS like protein is clustered together with an major facilitator superfamily transporter (An09g00190), whose expression is also downregulated significantly in CitB#99. CimA and IPMS, together with homocitrate synthase, belong to the LeuA dimer superfamily [27]. To explore the role of this novel gene cluster, its overexpression was studied.

### Overexpression of *cimA*

To test whether the gene product of An09g00170 is involved in organic acid biosynthesis we have overexpressed the putative *cimA* gene under control of *A. niger gpdA* expression signals. Upon transformation, 96 colonies were randomly picked from transformation plates, cultivated in microtiter plates and the supernatant analyzed on HPLC. Out of the tested 96 colonies, the strains CimA A10, CimA B3 and CimA D11 produced a compound with the same HPLC profile as CM and colony PCR confirmed the presence of pABgpdI-cimA (Additional file 1: Tables S2 and S3; PCR data not shown).

**Fig. 3:**
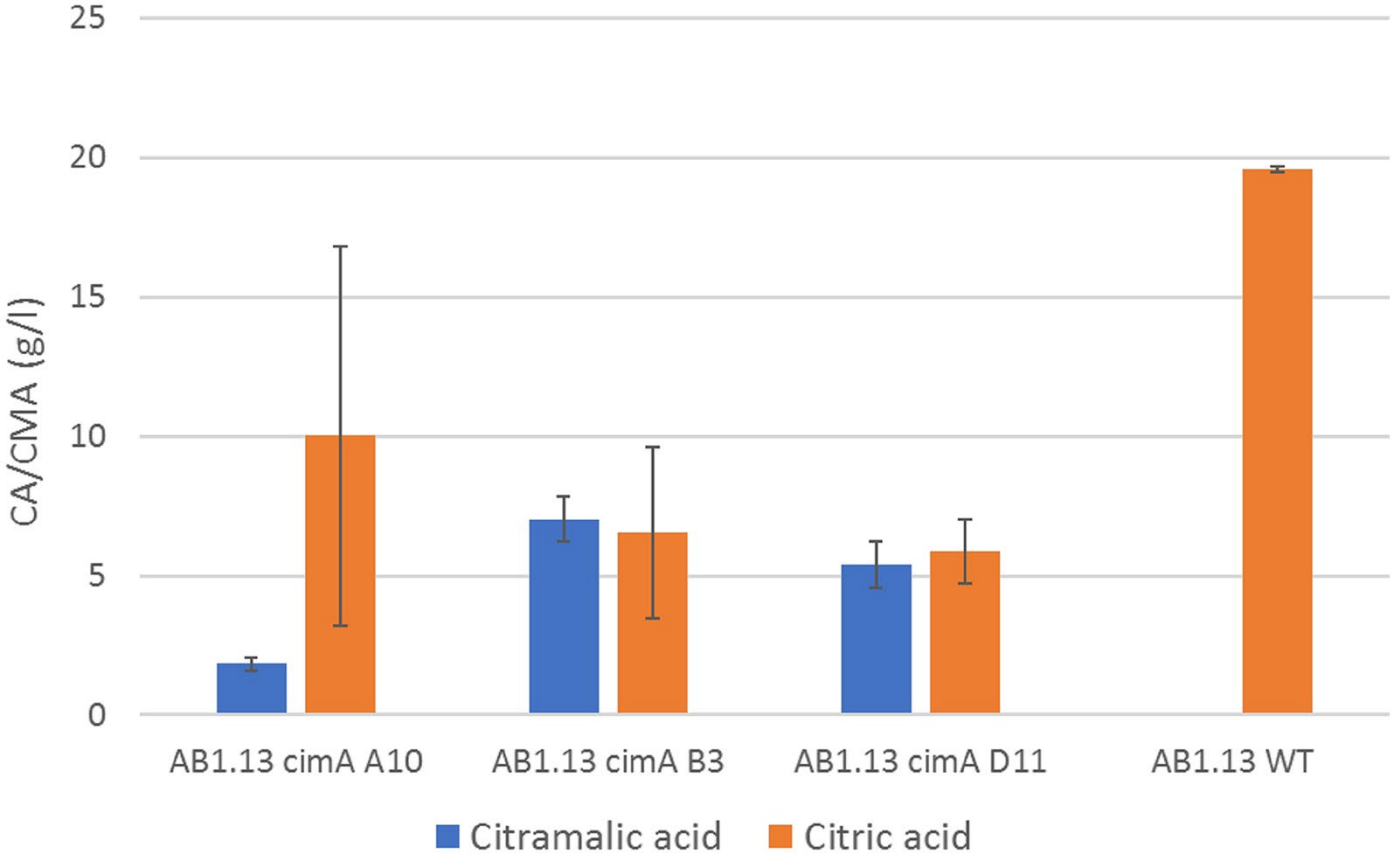
Shake flask cultivation of *cimA* overexpressing strains. Experiments were performed in duplicate. Samples were taken and measured after 280 h incubation

CM production was further tested in *cimA* overexpressing strains CimA A10, CimA B3 and CimA D11. Non baffled shake flasks were inoculated and samples taken for HPLC measurement. After 280 h of incubation CimA A10 had accumulated 1.83 g/l CM and 10.01 g/l CA, CimA B3 accumulated 7.03 g/l CM and 6.83 g/l CA, CimA D11 accumulated 5.41 g/l CM and 5.87 g/l CA, whereas the parental AB1.13 strain accumulated 19.55 g/l CA and no detectable CM (Fig. 3). These results indicate that the gene product of An09g00170 is involved in citramalate biosynthesis. To further boost the production of CM we have overexpressed the MFS multidrug transporter that is clustered together with *cimA* in the *A. niger* genome.

### Overexpression of *mfsB*

Having established CM production upon *cimA* overexpression, we subsequently tested the effect of overexpressing *mfsB* in CM producing strain CimA B3. For this purpose auxotrophic (*pyrE*−) strain was generated by cultivation on 5-fluoro-orotic acid. Transformation of CimA B3 *pyrE*− with *mfsB* expression cassette resulted in 21 transformants that were verified by PCR analysis (data not shown). Ten strains were selected for shake flask cultivation together with the parental CimA B3 strain to evaluate CM production (Table 4). Four transformants performed better in CM yield and titer compared with CimA B3 (CimA + MfsB #17, #27, #28, #85). The two best performing strains CimA + MfsB #27 and #28 were selected for further experiments.

**Table 4:** CM yield and titer of *mfsB* overexpressing strains

Strain	Titer	Yield
	g/l	g/g glucose
AB1.13 cimA B3 parent	2.71	0.07
AB1.13 cimA + mfsB 12	0.81	0.02
AB1.13 cimA + mfsB 13	1.04	0.02
AB1.13 cimA + mfsB 65	1.82	0.05
AB1.13 cimA + mfsB 59	1.99	0.06
AB1.13 cimA + mfsB 54	2.11	0.06
AB1.13 cimA + mfsB 102	2.15	0.06
AB1.13 cimA + mfsB 85	3.10	0.09
AB1.13 cimA + mfsB 17	3.21	0.09
AB1.13 cimA + mfsB 27	3.36	0.10
AB1.13 cimA + mfsB 28	3.87	0.11

### Shake flask cultivation

Overexpression of *mfsB* in CM producing CimA B3 strain resulted strains with increased CM yield. The CM production performance of two of these strains, CimA + MfsB #27 and #28, was compared with the parental CimA B3 and AB1.13 strains in 500 ml non-baffled shake flask cultivations. Strain AB1.13 produced no detectable CM, while max. 12.4 g/l CA was produced after 236 h, after which CA titers strongly reduced (Fig. 5a). This effect is caused by the depletion of glucose in the medium after 236 h (Fig. 5b). CM production of strains CimA B3, CimA + MfsB #27 and CimA + MfsB #28 is comparable between the three strains and final titers of 6.6 g/l, 6.4 g/l and 5.9 g/l CM is produced respectively after 333 h of cultivation (Fig. 5a). CA is also produced as side product in cultivations with strain CimA B3 (max. 9.2 g/l), CimA + MfsB #27 (max. 6.1 g/l) and CimA + MfsB #28 (max. 3.7 g/l) after 333 h. Interestingly, CA production in *mfsB* overexpressing strains only starts after 142 h of cultivation, whereas CA titer of 2.4 g/l is already achieved after 72 h in cultivation with strain CimA B3 (Fig. 5a). Equally interesting is the observation that glucose is only depleted in cultivations with strains AB1.13 and CimA B3 but not in cultivations with strains CimA + MfsB #27 and #28 with 24.9 g/l and 45 g/l glucose left respectively. This observation is also in line with the increased CM yield of strains CimA + MfsB #27 and #28 (Table 5). More detailed analysis of the HPLC results from flask cultivations of strains expressing both *cimA* and *mfsB* also identified a compound with HPLC characteristics similar to citraconic acid (Fig. 5).

**Table 5:** Yield and titer of CM and CA production by various *A. niger* strains

Strain	Citramalic acid		Citric acid	
	Max titer (g/l)	Max yield (g/g glucose)	Max titer (g/l)	Max yield (g/g glucose)
AB1.13 WT	0	0	12.35	0.14
CimA B3	6.62	0.07	9.19	0.10
CimA + MfsB 27	6.35	0.10	6.06	0.08
CimA + MfsB 28	5.87	0.11	3.73	0.07

In conclusion, by overexpressing *cimA* and *mfsB* we have converted *A. niger* into a system that predominantly produces CM (and eventually its degradation product citraconic acid) and reduced CA levels (Table 5).

## Discussion

Heterologous IA bioproduction in *A. niger* resulted in high levels of unexpected metabolic rewiring, as exemplified by the induction of two genes, *ictA* and *ichA,* that are involved in IA degradation upon high IA titers [5]. The proteins encoded by these genes intracellularly convert IA into a previously unknown compound. In this study we identified CM as being the end product of the IA biodegrading pathway in *A. niger*. We have shown that IA is converted into CM during IA biodegradation, by action of IctA and IchA as the genes encoding these enzymes are strongly induced upon IA bioproduction [10]. This is in contrast with the end products of the IA biodegrading pathways in *Y. pestis, P. aeruginosa* and *A. terreus*, which are pyruvate and acetyl-CoA. Surprisingly, the bacterium *Alcaligenes xylosoxidans* has also been reported to intracellularly convert IA into CM, indicating that *A. niger* is not the only organism with this phenotype [29]. IA degradation and concomitant CM bioproduction cessate by knocking out either *ictA* or *ichA* [10].

What the role of CM is in *A. niger* metabolism and why *A. niger* converts IA into CM is not yet clear. One explanation for the secretion of CM in a IA overproducing strain could be that the gene encoding the last step in the IA biodegrading pathway, *cclA* which codes for citramalyl-CoA lyase, is not induced in *A. niger* upon IA biodegradation and the conversion to pyruvate and acetyl-CoA therefore does not occur in *A. niger,* being a natural non-IA producing host [10]. Interestingly, Meijer et al. [30] have also detected citramalate in *A. niger*, however, this was in cell lysates of WT *A. niger* where normally the IA degradation pathway is not induced [10, 31]. This suggests that there must be other endogenous biosynthesis pathways for CM whose function is yet unknown. To explore possible novel organic acid biosynthesis pathways in *A. niger* we have looked into metabolic pathway rewiring in transcriptome data (Table 2). Interestingly, in this dataset we have observed the downregulation of a putative IPMS An09g00170, which upon overexpression drives CM production. This result prompted us to designate An09g00170 as citramalate synthase *cimA*. Furthermore, in our transporter comparison analysis, we have seen that the ortholog of the functionally characterized IA transporter from *U. maydis,* Itp1, is the characterized citrate transporter in *A. niger,* CexA (Table 3), while the putative ortholog to the functionally orthologous *A. terreus* MfsA is An02g07580. These results suggest that these organic acid transporters show significant redundancy, also explaining that without co-expression of a pathway specific transporter the related organic acid can still be exported albeit at low(er) levels [8, 15, 22, 32].

Citramalate synthase has been described as an enzyme from the archaea *M. jannaschii* that is a part of the isoleucine biosynthesis pathway and together with IPMS belongs to the LeuA dimer superfamily [26, 27]. Whereas IPMS catalyzes the condensation of acetyl-CoA with α-ketoisovalerate to form isopropylmalate in the leucine biosynthesis pathway, citramalate synthase catalyzes the condensation of acetyl-CoA with pyruvate to form citramalate [26, 27]. To the best of our knowledge, this is the first example of CimA driven CM production in filamentous fungi. We are still speculating about the role of CM in the metabolism of *A. niger*, however, it is possible that CM is an intermediate in the isoleucine biosynthesis pathway as is the case in archaea, and is clearly a topic for further research.

It is also interesting to note that *cimA* is clustered together with an major facilitator superfamily transporter An09g00190, which we have termed *mfsB*. This observation led us to speculate that *mfsB* is responsible for or involved in the cellular export of citramalate. Previously, it has been shown that the cellular export of metabolites can be the limiting factor resulting in low titers and yields [8, 32]. However, upon overexpression of *mfsB* we have not observed strongly increased titers of CM, but we have observed an increased CM yield and secretion of citraconate (Table 5). Moreover, during IA biodegradation and concomitant CM production, the expression of *mfsB* is strongly repressed (Table 2, Fig. 2) [10], suggesting that MfsB may not be the only CM transporter. At this point we do not have indications of which other exporter might serve this function.

Upon *mfsB* overexpression, the titer and yield of CA dropped, suggesting a change in the metabolism where CA production is reduced to favor CM production. As also citraconate is produced after prolonged cultivation upon *mfsB* overexpression, other metabolic conversion may take place driven by transporter action. This result further shows the crucial role of these transporters in organic acid production as is also recently shown by Wierckx et al. [33]. It is further interesting to note that also CA secretion resumed later on during cultivation by strains CimA + MfsB #27 and #28 (Fig. 5a). We speculate that a (nutrient) limitation in the cultivation medium may be causing this phenotype. This result would then indicate that medium optimization towards optimal CM production in *A. niger* is required. We have recently successfully performed medium optimization towards improved heterologous IA production in our lab [9]. Apart from medium optimization, genetic engineering to further optimize the CM biosynthesis may also be applied. We hypothesize that the overexpression of ATP-citrate lyase would improve the biosynthesis of CM by increasing the precursor pool of acetyl-CoA for CimA, similar as observed for IA bioproduction [9].

**Fig. 4:**
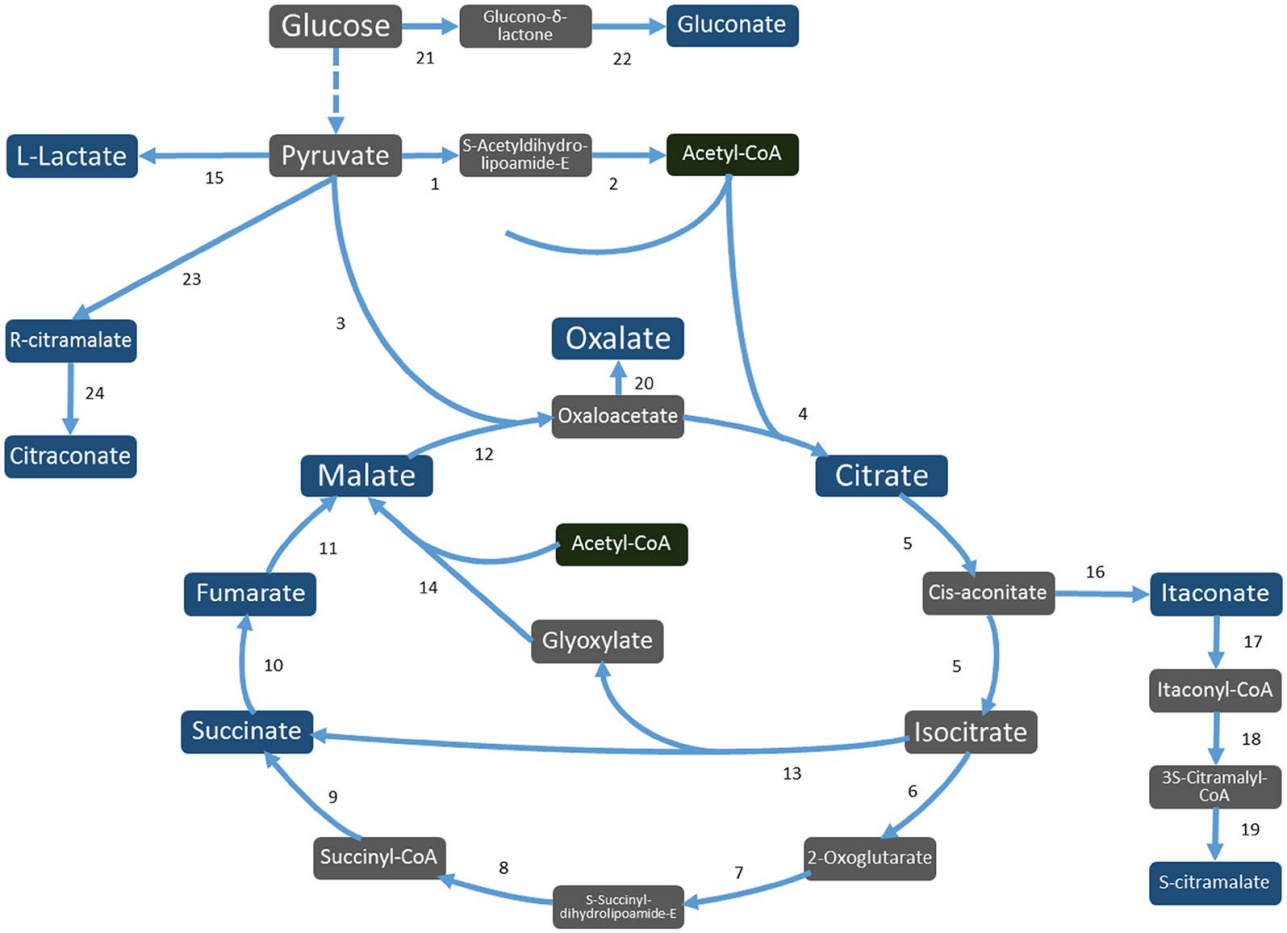
Putative organic acid biosynthesis pathways in *A. niger*. The enzymes facilitating the biochemical conversions are given with numbers and are linked to Table 2. Industrially relevant organic acids are indicated in dark blue. Figure adapted from Li et al. [13]

**Fig. 5:**
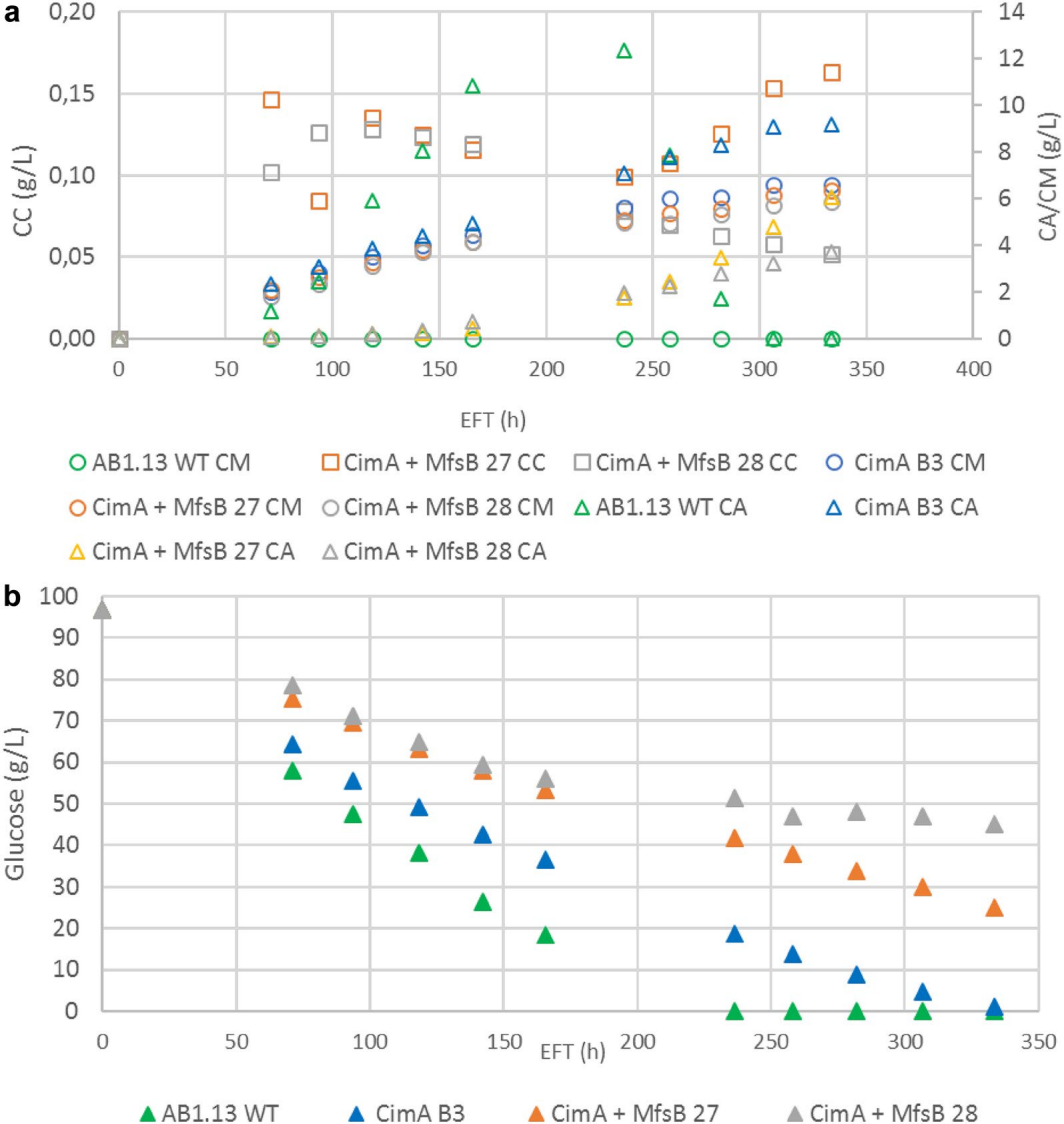
Shake flask experiment to compare the organic acid production of *cimA* and *mfsB* overexpressing strains with WT strain. **a** Production of CM, citric acid (CA) and citraconic acid (CC). **b** Consumption of glucose

It is also relevant to mention that the two CM biosynthesis pathways, as identified in our research, would produce two different enantiomers of CM (Fig. 4). The further elucidation of these pathways and the pathway-specific enantiomer that is produced is topic for further research, however the fact that in *cimA*/*mfsB* overexpression strains the produced CM seems to be converted further into citraconic acid (Fig. 5; Additional file 1: Tables S2 and S3) suggests that in that case R-citramalate is produced [34], while in the itaconic acid degradation pathway this can only be S-citramalate [35]. Moreover, CM is an interesting compound from industrial perspective, due to its potential to serve as bio-based precursor for methyl methacrylate synthesis, which in turn is the building block for acrylic glass (Plexiglas) [36, 37]. This has spurred further research activities into optimizing the bioproduction of CM [38].

## Conclusion

We have previously reported the intracellular biodegradation pathway of IA in *A. niger*. In this study we have identified the end product of this biodegradation pathway as being CM. Knock-out of the biodegradation pathway specific genes *ictA* or *ichA* results in the cessation of IA biodegradation and concomitant CM production. Furthermore, in this study we have identified, through transcriptome analysis, an alternative citramalate biosynthesis pathway, which upon overexpression drives bioproduction of citramalate in *A. niger*. The biosynthetic citramalate synthase is clustered with a putative transporter, which upon overexpression results in almost 2-fold higher citramalate yield on glucose, suggesting it to be a citramalate exporter. However, as also citraconate is secreted, these observations would require additional research similar as was recently done for the IA transporters [22, 33].

## Supplementary information

**Additional file 1: Table S1.** List of primers used in this study. **Table S2.** Organic acid references used on HPLC, their retention times on UV and RI detector and UV210_nm_/RI ratio. **Table S3.** HPLC data of samples from AB1.13 WT, CimA B3, CimA+MFSB #27 and CitB#99.

## Data Availability

Transcriptome data will be uploaded on GEO.

## Abbreviations

CC: citraconate
CA: citric acid
CM: citramalate
Cyto: cytosolic
DO: dissolved oxygen
GRAS: generally regarded as safe
HPLC: high performance liquid chromatography
IA: itaconic acid
LCM: liquid complete medium
Mito: mitochondrial
MM: minimal medium
MTP: microtiter plate
PCR: polymerase chain reaction
Per: peroxisomal
5′-FOA: 5′-fluoroacetic acid
