# Supplementary material for: Identification of novel citramalate biosynthesis pathways in Aspergillus niger
Source: Fungal Biol Biotechnol. 2019 Nov 19;6:19. doi: 10.1186/s40694-019-0084-7 (PMC6862759; doi:10.1186/s40694-019-0084-7)
Supplement: Supplementary file 1 — Additional file 1: Table S1. List of primers used in this study. Table S2. Organic acid references used on HPLC, their retention times on UV and RI detector and UV210nm/RI ratio. Table S3. HPLC data of samples from AB1.13 WT, CimA B3, CimA+MFSB #27 and CitB#99. [file 40694_2019_84_MOESM1_ESM.docx]

| **Primer number (#)** | **Name** | **Sequence 5’ → 3’** |
| --- | --- | --- |
| **80** | pABgpd-for-sfil | TCACTATAGGGCGAATTGGC |
| **81** | pABgpd-rev-sfil | CTGGAAAGCGGGCAGTGA |
| **98** | WBT1,141 R | CTCTTCAGGCCCCTATGTAT |
| **99** | WBT 46 F | GAGGTGATTGAGTGTGGGAC |
| **143** | pABgpd-2,028 F | TCGTTGCGTCAGTCCAGA |
| **329** | Qdr3_seqprimer3-rev | TTAGTCACCACCGCAGCATT |
| **331** | cimA-2,679 R | CGTGAAATCGAACTCGGTCT |
| **406** | A. niger acl2 R | AAGGGCTCAACCAGGAAC |
| **430** | mfsB ORF SEQ1 F | TATTACGACCCGTGAAGATC |
| **431** | mfsB ORF SEQ2 F | TTTCCACTTGAACGAGCC |
| **432** | mfsB F + NcoI/EcoRI | GAATTCCCATGGCAGCAGATCAGAAC |
| **433** | mfsB R + BglII/EcoRI | GAATTCAGATCTACCCGTATGTAGGACTGGTC |
| **488** | cimA Fw southern | GCCATATTTACCAATGGATCC |
| **489** | cimA Rv southern | TTGGAGAAGAAGCTGTTGACC |

**Additional file 1: Table S1: Primers used in this study.**

|  | **Citrate (CA) standard 7 g/L** | | |
| --- | --- | --- | --- |
|  | **RT** | **Area** | **210/RI** |
| UV210 | 7,771 | 6623188 | 0,63 |
| UV270 | 7,777 | 11480 |  |
| RI | 8,074 | 10508899 |  |
|  |  |  |  |
|  | **Citramalate (CM) standard 1 g/L** | | |
|  | **RT** | **Area** | **210/RI** |
| UV210 | 9,48 | 863748 | 0,59 |
| UV270 | 9,482 | 1817 |  |
| RI | 9,789 | 1459400 |  |
|  |  |  |  |
|  | **Cis-aconitate (CAA) standard 0,5 g/L** | | |
|  | **RT** | **Area** | **210/RI** |
| UV210 | 7,307 | 13843912 | 27,74 |
| UV270 | 7,299 | 373576 |  |
| RI | 7,614 | 499103 |  |
|  |  |  |  |
|  | **Trans-aconitate (TAA) standard 0,5 g/L** | | |
|  | **RT** | **Area** | **210/RI** |
| UV210 | 9,448 | 39047506 | 30,76 |
| UV270 | 9,439 | 1571443 |  |
| RI | 9,755 | 1269306 |  |
|  |  |  |  |
|  | **Itaconate (IA) standard 0,7 g/L** | | |
|  | **RT** | **Area** | **210/RI** |
| UV210 | 12,129 | 30911479 | 27,72 |
| UV270 | 12,122 | 83769 |  |
| RI | 12,435 | 1115002 |  |
|  |  |  |  |
|  | **Citraconate (CC) standard 1 g/L** | | |
|  | **RT** | **Area** | **210/RI** |
| UV210 | 10,68 | 51799936 | 35,89 |
| UV270 | 10,673 | 837817 |  |
| RI | 11 | 1443219 |  |
|  |  |  |  |
|  | **Pyruvate (PA) standard 1,4 g/L** | | |
|  | **RT** | **Area** | **210/RI** |
| UV210 | 9,75 | 10063903 | 5,07 |
| UV270 | 9,742 | 168420 |  |
| RI | 10,051 | 1984217 |  |

**Additional file 1: Table S2: Organic acid references used on HPLC, their retention times on UV and RI detector and UV210_nm_/RI ratio.**

|  | **Sample AB1.13 WT CA** | | |  |  | **Sample AB1.13 WT TAA** | | |  |  |  |  |  |
| --- | --- | --- | --- | --- | --- | --- | --- | --- | --- | --- | --- | --- | --- |
|  | RT | Area | 210/RI |  |  | RT | Area | 210/RI |  |  |  |  |  |
| UV210 | 7,762 | 227539 | 0,73 |  | UV210 | 9,447 | 153971 | 24,56 |  |  |  |  |  |
| UV270 | 7,766 | 971 |  |  | UV270 | 9,44 | 6620 |  |  |  |  |  |  |
| RI | 8,066 | 311729 |  |  | RI | 9,713 | 6268 |  |  |  |  |  |  |
|  |  |  |  |  |  |  |  |  |  |  |  |  |  |
|  | **Sample CimA B3 CA** | |  |  |  | **Sample CimA B3 CM** | | |  |  |  |  |  |
|  | RT | Area | 210/RI |  |  | RT | Area | 210/RI |  |  |  |  |  |
| UV210 | 7,757 | 977434 | 0,65 |  | UV210 | 9,464 | 631003 | 0,60 |  |  |  |  |  |
| UV270 | 7,76 | 2998 |  |  | UV270 | 9,439 | 11609 |  |  |  |  |  |  |
| RI | 8,067 | 1495236 |  |  | RI | 9,767 | 1058658 |  |  |  |  |  |  |
|  |  |  |  |  |  |  |  |  |  |  |  |  |  |
|  | **Sample CimA+MfsB #27 CA** | | |  |  | **Sample CimA+MfsB #27 CM** | | |  |  | **Sample CimA+MfsB #27 CC** | | |
|  | RT | Area | 210/RI |  |  | RT | Area | 210/RI |  |  | RT | Area | 210/RI |
| UV210 | 7,759 | 396399 | 0,64 |  | UV210 | 9,463 | 582820 | 0,92 |  | UV210 | 10,675 | 790552 | 12,19 |
| UV270 | 7,777 | 873 |  |  | UV270 | 9,439 | 13866 |  |  | UV270 | 10,688 | 12725 |  |
| RI | 8,067 | 621156 |  |  | RI | 9,777 | 636460 |  |  | RI | 10,954 | 64859 |  |
|  |  |  |  |  |  |  |  |  |  |  |  |  |  |
|  | **Sample CitB#99 IA** | |  |  |  | **Sample CitB#99 CM** | |  |  |  |  |  |  |
|  | RT | Area | 210/RI |  |  | RT | Area | 210/RI |  |  |  |  |  |
| UV210 | 12,114 | 32684837 | 27,82 |  | UV210 | 9,438 | 429797 | 0,75 |  |  |  |  |  |
| UV270 | 12,106 | 85087 |  |  | UV270 |  |  |  |  |  |  |  |  |
| RI | 12,42 | 1175006 |  |  | RI | 9,746 | 574937 |  |  |  |  |  |  |

**Additional file 1: Table S3:** **HPLC data of samples from AB1.13 WT, CimA B3, CimA+MFSB #27 and CitB#99.**
